# Microbial communities of the Lemon Creek Glacier show subtle structural variation yet stable phylogenetic composition over space and time

**DOI:** 10.3389/fmicb.2015.00495

**Published:** 2015-05-20

**Authors:** Cody S. Sheik, Emily I. Stevenson, Paul A. Den Uyl, Carli A. Arendt, Sarah M. Aciego, Gregory J. Dick

**Affiliations:** ^1^Department of Earth and Environmental Sciences, University of Michigan, Ann Arbor, MIUSA; ^2^Ecology and Evolutionary Biology, University of Michigan, Ann Arbor, MIUSA; ^3^Center for Computational Medicine and Bioinformatics, University of Michigan, Ann Arbor, MIUSA

**Keywords:** microbial ecology, geomicrobiology, glaciers, diversity, temporal dynamics

## Abstract

Glaciers are geologically important yet transient ecosystems that support diverse, biogeochemically significant microbial communities. During the melt season glaciers undergo dramatic physical, geochemical, and biological changes that exert great influence on downstream biogeochemical cycles. Thus, we sought to understand the temporal melt-season dynamics of microbial communities and associated geochemistry at the terminus of Lemon Creek Glacier (LCG) in coastal southern Alaska. Due to late season snowfall, sampling of LCG occurred in three interconnected areas: proglacial Lake Thomas, the lower glacial outflow stream, and the glacier’s terminus. LCG associated microbial communities were phylogenetically diverse and varied by sampling location. However, Betaproteobacteria, Alphaproteobacteria, and Bacteroidetes dominated communities at all sampling locations. Strict anaerobic groups such as methanogens, SR1, and OP11 were also recovered from glacier outflows, indicating anoxic conditions in at least some portions of the LCG subglacial environment. Microbial community structure was significantly correlated with sampling location and sodium concentrations. Microbial communities sampled from terminus outflow waters exhibited day-to-day fluctuation in taxonomy and phylogenetic similarity. However, these communities were not significantly different from randomly constructed communities from all three sites. These results indicate that glacial outflows share a large proportion of phylogenetic overlap with downstream environments and that the observed significant shifts in community structure are driven by changes in relative abundance of different taxa, and not complete restructuring of communities. We conclude that LCG glacial discharge hosts a diverse and relatively stable microbiome that shifts at fine taxonomic scales in response to geochemistry and likely water residence time.

## Introduction

As primary agents of erosion (Hallet et al., 1996), glaciers represent a vast source of freshly mined, crushed, and weathered bedrock material. Downstream deposition of this mineral flour, especially in marine environments, can potentially stimulate productivity via iron or silicate fertilization (Falkowski et al., 1998; Bhatia et al., 2013). Thus, glaciation has been implicated in driving Earth’s climate on millennial time scales (Walker et al., 1981). Until recently the subglacial environment, where freshly milled rock flour is chemically weathered, was considered devoid of microorganisms (Skidmore et al., 2005) and weathering processes were thought to be driven solely by abiotic reactions (Raiswell, 1984; Tranter et al., 1993). It is now understood that subglacial environments are teeming with diverse, abundant, and active microbial communities (Sharp et al., 1999; Skidmore et al., 2000, 2005; Boyd et al., 2010; Hamilton et al., 2013). Molecular studies routinely recover sequences with high similarity to cultivated microorganisms capable of mediating chemical and whole mineral weathering processes such as sulfur and iron oxidation (Skidmore et al., 2005; Hamilton et al., 2013; Mitchell et al., 2013). Incubation studies indicate that subglacial microorganisms contribute significantly to the chemical weathering process (Montross et al., 2013; Boyd et al., 2014) and are influenced by bedrock composition (Skidmore et al., 2005; Mitchell et al., 2013). Furthermore, microbial respiration may enhance carbonation reactions, resulting in greater dissolution of silicates (Wadham et al., 2010). These microbial driven processes are most apparent in the visually striking case of Blood Falls, Antarctica, where microbial activity in brines beneath Taylor Glacier drives sulfur and iron cycling, resulting in the discharge of iron (II), which is then oxidized as fluids are discharged from the glacial terminus (Mikucki and Priscu, 2007; Mikucki et al., 2009). Thus it is apparent that low-temperature, microbe–lithosphere interactions are pervasive in both modern glaciers and ice-sheets, and likely played important biogeochemical roles throughout Earth history.

Given the implications of an active subglacial microbiome on weathering processes, much attention has been placed on understanding the composition and geochemical role of these microbial communities (Sharp et al., 1999; Skidmore et al., 2000, 2005; Mikucki and Priscu, 2007; Boyd et al., 2010, 2011; Hamilton et al., 2013; Dieser et al., 2014). However, because of the difficulties in obtaining sediment and water samples directly from the subglacial environment, a majority of studies focus on outflows from the terminus of the glacier. During the melt season the terminus undergoes drastic physical changes; warming temperatures drive the melting of snow cover, exposed ice (basal and surface), and potentially subglacial, seasonally frozen sediments (Fountain and Walder, 1998; Hodson et al., 2008). The geochemistry of subglacial outflow waters reflects these changes; as outflow volumes increase, dilution of solutes is observed (Tranter et al., 1997; Skidmore and Sharp, 1999; Anderson, 2007). Furthermore, during extreme melting, channels connecting the surface to subglacial environments may develop (Nienow et al., 1998; Tranter, 2005; Hodson et al., 2008), thereby delivering nutrients such as labile carbon (Hodson et al., 2008), nitrogen (Tranter et al., 1994), and potentially microbial communities to the subglacial environment and out through the glacial terminus. Thus, discharged microbial communities could vary highly throughout the melt season depending on the interconnectivity of the surface and subglacial systems.

**FIGURE 1:**
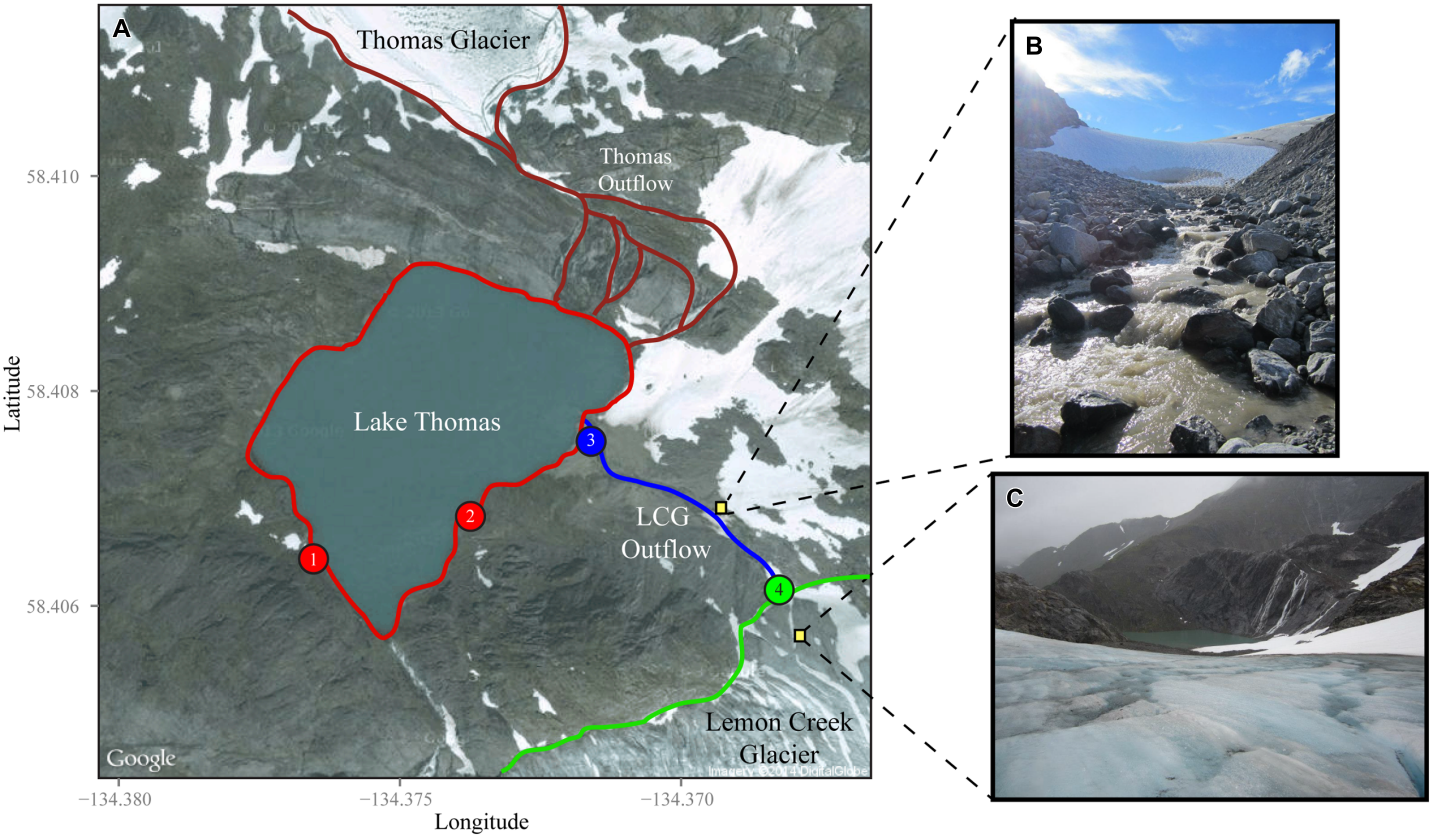
**Overview of sampling locations at Lemon Creek Glacier (LCG). (A)** Satellite image taken from Google Maps shows sampling locations, Lake Thomas (red), LCG outflow and runoff (blue), and LCG terminus outflow (green). Numbers within the filled circles correspond to sampling locations in **Figures 2–4**. Late season pictures from the site show the outflow stream from the glacier terminus **(B)** and the catchment lake taken from atop the glacier **(C)**. For scale the latitude tick marks represent approximately 222 m.

In view of the limited number of subglacial microbial ecology studies, and given that the underlying glacial bedrock can greatly influence the microbial community’s chemosynthetic potential, we sought to (I) characterize the composition of microbial communities associated with the glacial terminus, (II) track how these communities vary over time, and (III) investigate whether microorganisms in glacial discharge waters are correlated with the subglacial geochemistry. To answer our questions, we routinely sampled from Lemon Creek Glacier (LCG) during the early and peak melt season of 2012. LCG is a small valley, land-terminating glacier located ~6.5 km northeast of Juneau in southern Alaska (Heusser and Marcus, 1960). Due to its proximity to the Gulf of Alaska, LCG has a maritime climate. LCG is a south to north flowing glacier where the primary glacial outflow flows to the north and drains into proglacial Lake Thomas (see **Figure 1**). LCG also has a secondary, low-volume outflow, to the west, that feeds a lower proglacial lake. Since monitoring began in 1953, LCG has undergone a significant reduction in ice thickness of ~22 m and has retreated ~800 m (Miller and Pelto, 1999). LCG resides on the mid-Cretaceous central pluton-gneiss belt (Gehrels and Berg, 1994) with late Permian high-grade metamorphic and volcanic rock surrounding the sampling site. Stevenson et al. (under review), the first geochemical characterization of LCG, indicates secondary, chemical weathering processes are occurring at LCG, as suspended outflow sediments are minerologically similar but isotopically differentiated over time.

Using a high throughput, 16S rRNA tagged sequencing approach, we show that the membership of microbial communities were quite stable over the course of sampling. Microbial community structure varied and was correlated with time, which was a proxy for geographical location (proglacial lake, outflow stream, and subglacier terminus outflow) and sodium concentration in our study. However, the observed differences in microbial community structure were driven primarily by changes in OTU abundance rather than major phylogenetic restructuring.

## Materials and Methods

### Sampling Site, Collection, and Geochemical Measurements

Sampling was performed between the months of May and September of 2012 at LCG. The 2012 sampling season was unseasonable cold, resulting in a persistent snowpack, well through the summer melt season. The terminus of LCG was located at 58°24′22.14′′ N and 134°22′6.18′′ W (**Figure 1**). Terminus discharge rates were measured using an Acoustic Doppler Velocimeter Flow-tracker and ADV Flowtracker (Sontek, San Diego, CA, USA). Flow rates were not taken during periods of high discharge, which typically followed heavy rainfall events. Field geochemical measurements of temperature, dissolved oxygen, conductivity, and pH were taken with a YSI Pro Plus multiparameter meter (Yellow Springs, OH, USA). Alkalinity was quantified with Total Alkalinity Reagent (Fisher Scientific, Pittsburgh, PA, USA). For high alkalinity waters, 10 mL of alkalinity reagent were combined with 100 mL of filtered (0.22 μm) subglacial water, thoroughly mixed, and pH measured. For low alkalinity waters, 100 mL of filtered waters and 1 mL of alkalinity reagent were combined. The pH was then converted to the total alkalinity according to methods in (Hedin et al., 1994; Fujita et al., 2008).

Suspended particle filtering was done at near daily intervals at approximately the same time (early afternoon). Microbial samples were taken intermittently but immediately after geochemistry sampling. Twenty liters of waters with suspended particles (including microbes) were filtered through a 0.22 μm, 142 mm Durapore PVDF membrane filter (EMD Millipore, Billerica, MD, USA) using a peristaltic pump and custom metal free filter housing. For microbiology, filters were aseptically removed from filter housings, placed into 15 ml conical tubes, submerged with RNAlater (Ambion, Life Technologies, Grand Island, NY, USA) and kept at near freezing until shipment to the laboratory where filters were then frozen at -20°C.

A detailed description of the geochemical methodology and analysis of suspended metals and trace metals is provided in Stevenson et al. (under review). Briefly, sediment was removed from filters with Super-Q (SQ) water (Millipore, >18.2 MΩ), dried and digested (10 mg) for 7 days in concentrated nitric and hydrofluoric acids. Sediments were dried a second time and digested further for 24 h in *aqua regia*. Major and minor elements were quantified in triplicate (3 ml aliquots) by ICP-MS (ELEMENT2, Thermo Scientific). Only geochemical measurements where a coupled microbial sample was taken are presented in the current study.

### DNA Extraction, PCR Amplification, PCR Cleanup, and Pyrosequencing

DNA was extracted from ¼ of each filter, from both glacial samples and control filters, using the MoBio PowerSoil DNA isolation kit (Carlsbad, CA, USA) with modification to the bead beating procedure. Tubes were bead beat using the MP-Bio FastPrep-24 (Santa Ana, CA, USA) at a setting of 6.5 for 45 s. PCRs were performed in triplicate 25 μL reactions for each sample consisting of 12.5 μL 5Prime HotMasterMix (Gaithersburg, MD, USA), 1 μL each of forward and reverse primer (15 μM), 1 μL DNA, and 9.5 μL of PCR grade water. Two control PCRs were also included using “putative DNA” extracted from control filters and a DNA negative control consisting of PCR-grade water (Ambion). DNA concentrations ranged from 2 to 10 ng μL^-1^ across all samples. PCR primers targeted the V4 region (515F-806R) of the 16S rRNA gene (Bates et al., 2011) and contain a 454 sequencing adapter and a 12 bp error correcting barcode (Fierer et al., 2008). PCR consisted of initial denaturation 94°C for 4 min followed by 30 rounds of 94°C for 30 s, 50°C for 1 min, 72°C for 1 min, and a final extension step was included at 72°C for 10 min. Triplicate PCR reactions were pooled and cleaned with the MoBio UltraClean PCR cleanup kit. PCR products from each pooled and cleaned reaction was quantified by PicoGreen (Invitrogen, Life Technologies, Grand Island, NY, USA) and combined at equal concentrations. Pyrosequencing was performed by the laboratory of Dr. Vincent Young at the University of Michigan with 454-titanium chemistry (454 Life Sciences, A Roche Company, Branford, CT, USA). Sequences may be obtained from the Sequence Read Archive SRR1296545.

### DNA Read Processing and Statistics

Qiime v. 1.6.0 software (Caporaso et al., 2010) was used to parse pyrosequencing reads by barcode, and screen by quality with minlength = 180 and number of homopolymers set to eight. The majority (95%) of resulting reads were 250 bases in size. Reads were error corrected with Ampliconnoise (Quince et al., 2011). Operational taxonomic units (OTUs) were defined *de novo* with Uclust (Edgar, 2010) using a 0.97 cutoff and the following alterations to the default settings minsize = 1, optimal = T, and pick = T. Representative OTU sequences were picked based on highest abundance. Uchime (Edgar et al., 2011) was used to *de novo* detect and remove chimeric OTU sequences. All OTUs were classified to the Silva v.111 database (Pruesse et al., 2007) using the RDP naïve Bayesian method (Wang et al., 2007). To confirm the original classifications, a BLASTn of the top OTUs was performed using the Silva v.111 database. Before calculating alpha and beta diversity measures, the sequence library subsampled to a uniform depth of 1354 sequences per sample (based on the sample with the least number of sequences), and a phylogenetic tree was calculated with FastTree (Price et al., 2009). Alpha diversity metrics, Shannon, Simpson, Good’s coverage, and phylogenetic diversity were calculated in Qiime. Rare OTUs (<2 occurrences) were removed prior to calculation of Bray–Curtis dissimilarities. Beta diversity patterns were visualized with principal coordinate analysis (PCoA).

Alternative measures of phylogenetic distance, *SES.mpd* (SE size of mean pairwise distances) and *SES.mntd* (SE size of the mean nearest taxon distance), were calculated with the Picante package (Kembel et al., 2010) in R-Core-Team (2013). Correlations between geochemical measurements and microbial community structure were calculated using mantel tests. Geochemical distance matrices were calculated with vegdist using the vegan package (Oksanen et al., 2013) in R with the Bray–Curtis dissimilatory metric. Mantel tests utilizing Spearman product-moment correlation coefficient were performed with environmental distance matrices and Bray–Curtis derived microbial community patterns from PCoA analysis. In addition, an adonis model (analogous to a non-parametric MANOVA) was used to verify mantel tests and to assess whether changes in microbial communities and geochemistry were independent of sampling location. Beta dispersion analysis (betadisper in vegan) was used to test the intergroup homogeneity between sampling sites. Differences in homogeneity were tested using a tukey *post hoc* significance test in R.

## Results

### Geochemistry Varies by Time and Approximates Location of Sampling

Over the course of ~4 months, near daily geochemical measurements were taken from LCG (Stevenson et al., under review). However, measurements only coupled to microbial samples are presented here. Because of heavy snowfall covering the glacier and Lake Thomas (>4 m), early season, sampling began in lower and upper portions of Lake Thomas (**Figure 1**, sites 1 and 2). As snowfall melted, the LCG outflow/snow melt stream and terminus of the glacier were revealed and sampling was systematically shifted up the outflow stream to the glacial terminus (**Figure 1**, sties 3 and 4). Microbial sampling of Lake Thomas was not done later in the season. Concentrations of sodium, magnesium, potassium, and calcium were generally lower in the proglacial stream and terminus discharge compared to the proglacial lake, whereas pH, conductivity, alkalinity, and concentration of iron and aluminum displayed more variable trends (Supplementary Figure S1). Water temperature was consistently <1.0°C (data not shown), reflective of subglacial conditions. *In situ* measures of pH and conductivity showed that samples were circa neutral to alkaline (pH range of ~7–8.5) and low conductivity (5–25 μS). pH of the feeder stream and glacial terminus (mean: ~7.4) were lower than the lake (mean: ~7.9) but this difference was not significant (Supplementary Figure S2). Over time, which is approximate to sampling location, pH, alkalinity, magnesium, potassium, calcium, and conductivity all showed a strong and consistent trend, with increasing values in the lake from day 150 to 173, decreasing values in the stream from day 177 to 202, and increasing values in the glacier terminus from day 220 to 250 (Supplementary Figures S1 and S2). Sodium showed a more consistent decreasing trend throughout the sampling campaign with the exception of the last two sampling days. Potassium showed a similar trend to sodium but its decrease was less pronounced. Magnesium and calcium were relatively stable over time except for samples taken from the lake (Supplementary Figure S1). Despite a relative decrease in the concentration of all cations, ratios of di- to monovalent cations showed an increasing trend over the course of sampling (Supplementary Figure S3). The decreases in solute concentrations and conductivity observed from day 175 to 225 are typical for glacial outflows and are associated with outflow rates and water volume. Differences observed in catchment lake geochemistry over time are likely influenced by physical factors such as glacial melt discharge, mixing, depth, stratification, flux of surface snowmelt, and meteorological factors. This data shows strong patterns when divided by sampling sites, especially for sodium, magnesium, potassium, and calcium (Supplementary Figure S2).

### Taxonomy of Microbial Communities over Time and Space at Lemon Creek Glacier

**FIGURE 2:**
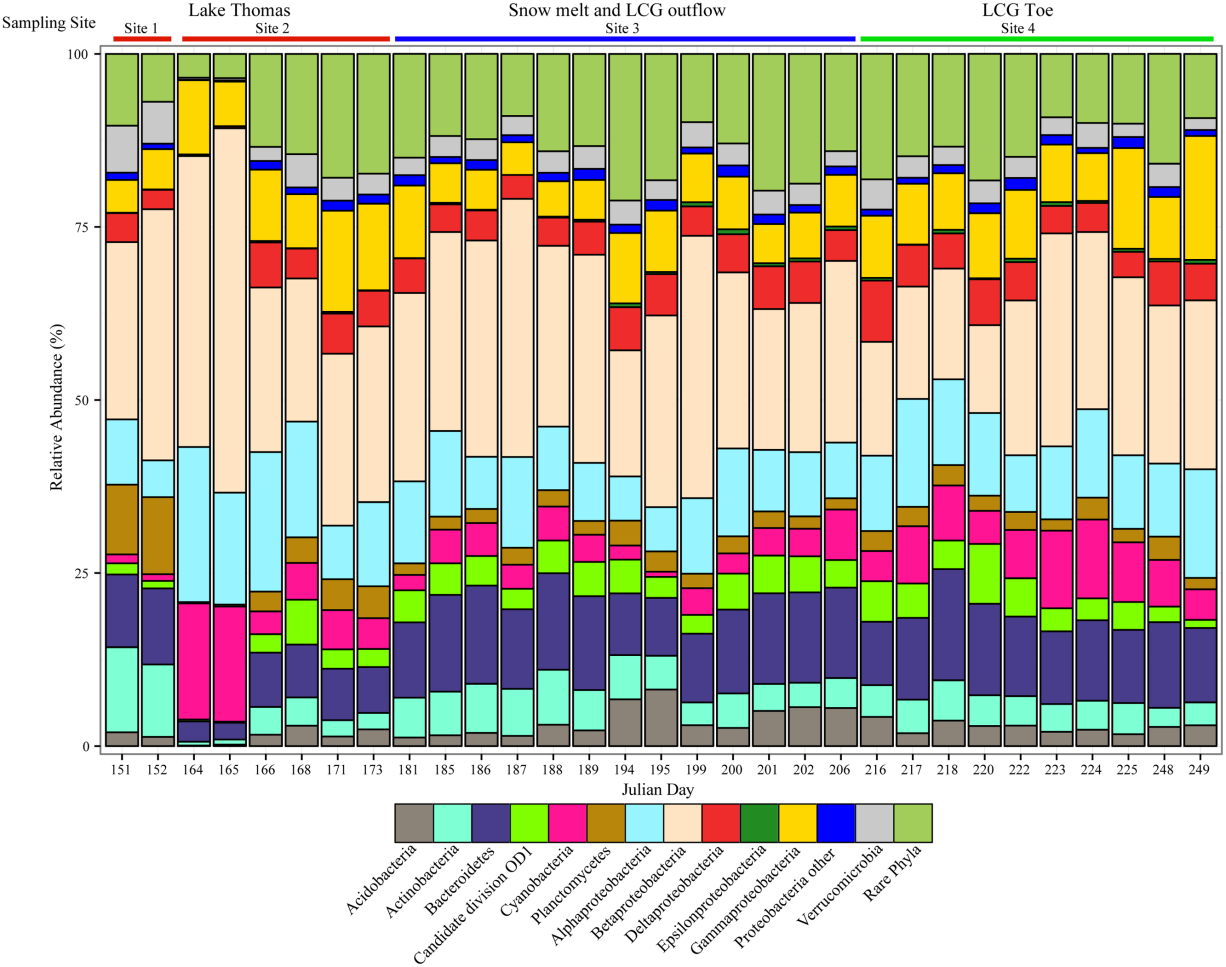
**Taxonomy of the top phyla and classes recovered in pyrosequencing libraries**. Samples are arranged in chronological order from early (left) to late season (right). Colored bars above the graph indicate approximate sampling location. Taxonomy of OTUs_0.97_ is based on the Silva v. 111 database.

At the phylum/class level, taxonomic distribution was relatively consistent across all samples. Proteobacteria were the most abundant phylum at all sampling times, followed by Bacteroidetes (**Figure 2**). Betaproteobacteria were the most abundant group in all samples, accounting for nearly 50% of the community in some samples. Alpha-, Gamma-, and Deltaproteobacteria were also abundant, rivaling, or in some cases surpassing Bacteroidetes. The abundance of Epsilonproteobacteria was low across all sampling sites and ranged from undetected to ~0.8% of the community. Several phyla typically found in anoxic systems were identified at low abundance, including BD1-5, OD1, OP11, OP3, Armatimonadetes, SR1, and Methanomicrobiales (encompassed by “rare phyla” in **Figure 2**). Archaea were detected in most samples and were associated primarily with Euryarchaeota and Thaumarchaeota. Because our PCR primers were not specific for Archaea, their diversity and abundance is likely under-represented.

**FIGURE 3:**
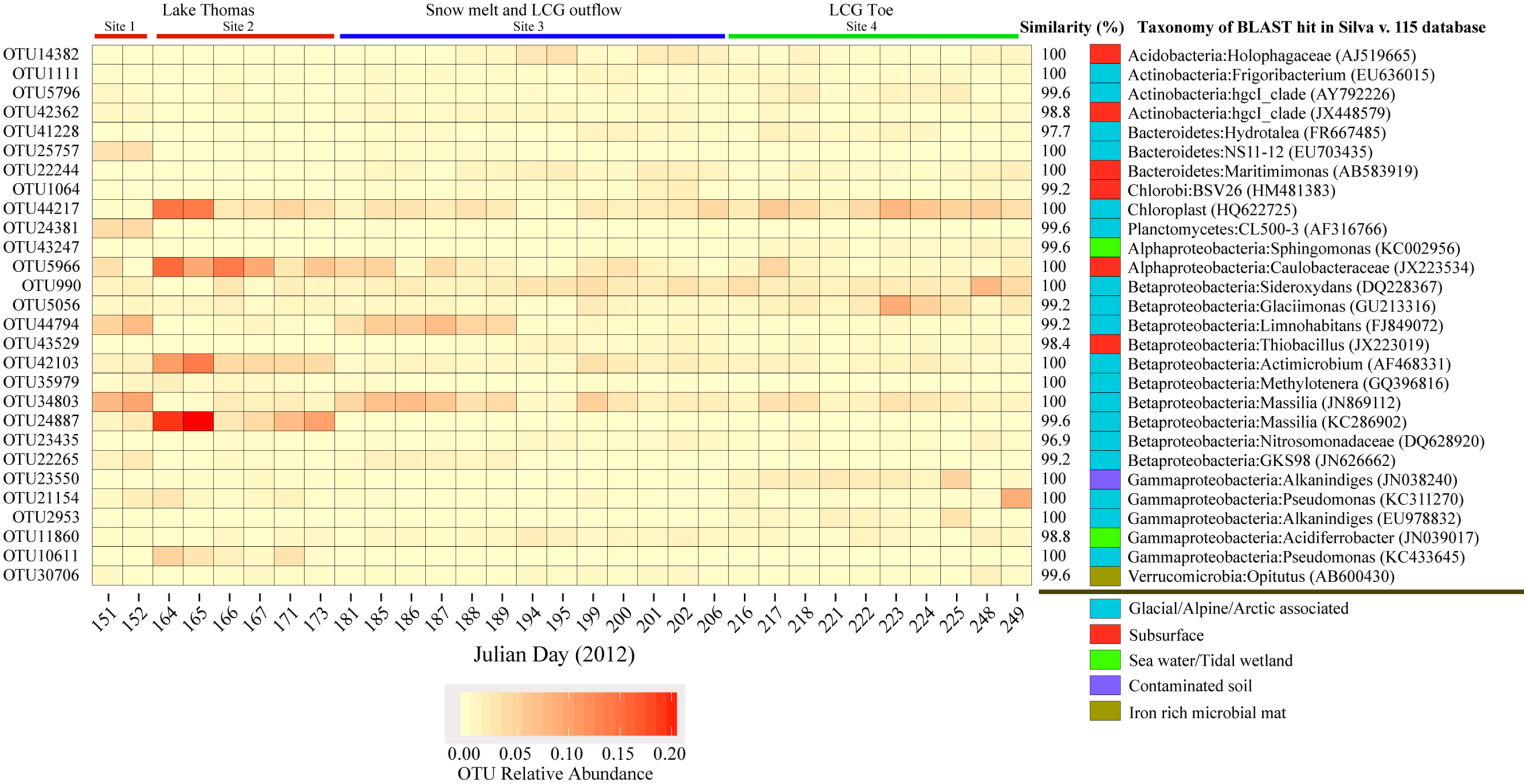
**Heatmap depiction of the relative abundance of the top OTUs, their putative taxonomy and closest match from Silva v. 111 NR SSU database**. Blast hits were chosen based on alignment length and similarity.

Analysis of the top OTUs revealed a dynamic landscape across time and space (**Figure 3**). Several highly abundant OTUs were dominant only at certain times and sampling locations (**Figure 3**). Of these top OTUs, a total of twenty were shared amongst all three sites. Most of these OTUs were highly similar to sequences from other glacial environments (ice, water, and snow cover) or from subsurface environments. Ten OTUs (from a total of 28) were not similar to sequences retrieved from glacial environments; these were mostly similar to sequences from soil, sediment, or surface seawater (see table in **Figure 3**). Several of these top OTUs were highly similar to organisms with chemolithoautotrophic or methylotrophic metabolism; i.e., OTU23435 (ammonia oxidation), OTU990 (iron oxidation; present in nearly all samples), OTU43529 (sulfur oxidation), and OTU35979 (methylotrophy). Finally, many of the top OTUs were similar to metabolic generalists such as *Pseudomonas*; these groups are generally metabolically diverse and versatile, thus it is difficult to infer their putative functions.

### Taxonomic Richness and Stability of Glacial Discharge Communities

**FIGURE 4:**
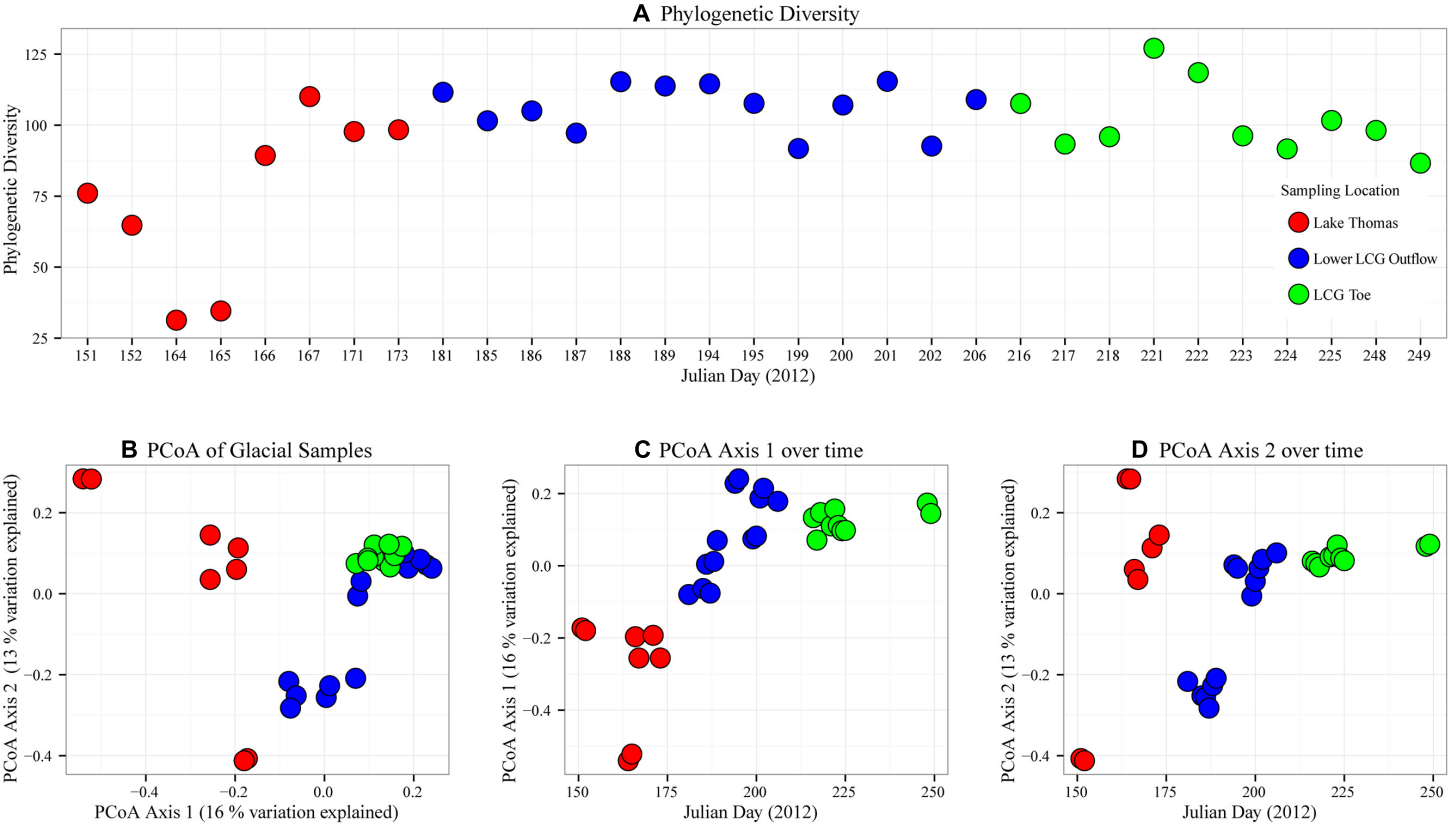
**Temporal microbial community diversity patterns at LCG**. Prior to calculation of alpha and beta diversity, rare OTUs (<2 occurrences) were removed and the OTU table was subsampled to a uniform depth of (1354 sequences). In all panels sampling location is indicated by color; lower Lake Thomas (red), lower outflow stream (blue), and glacial terminus (Green). **(A)** Temporal patterns of phylogenetic diversity (alpha diversity metric), **(B)** principal coordinate analysis (PCoA) assessing microbial community similarity (beta diversity), **(C,D)** temporal patterns of community similarity assessed using the first axis and second axis of the PCoA in panel **(B)**.

Observed species richness was consistently greater than 1000 OTUs (**Table 1**). After sub-sampling normalization, species richness (Shannon Index) and phylogenetic diversity were variable across sampling times (see SD in **Table 1**), but mean richness and coverage were higher for the transitional stream and glacial terminus. Because alpha diversity can be highly influenced by spurious OTUs (e.g., sequences that should have been incorporated into pre-existing OTUs but are excluded and form their own OTUs), phylogenetic diversity was calculated (Faith, 1992, 2013). We observed lower values of phylogenetic diversity in the initial pro-glacial lake and stream samples, but by Julian days 166–167 phylogenetic diversity increased and remained relatively stable across all sampling locations until the termination of sampling on Julian day 249 (**Figure 4A**).

**Table 1:** Summary of sequencing results and alpha diversity for each of the three sampling locations.

	Lower lake		Stream		Glacier toe	
	Mean	SD	Mean	SD	Mean	SD
Total Sequences	10050	6739.6	4183.4	862.4	2847.6	1329.9
Total OTUs	1784	617.3	1733.3	713.3	1190.4	477.9
Rarified No. Sequences	1354	–	1354	–	1354	–
Rarified No. OTUs	594.5	121.7	720.9	208.2	692.9	89.3
Shannon index	7.8	0.5	8.4	1.4	8.5	0.4
Phylogenetic diversity	89.4	15.3	99.2	27.1	99.8	10.1
Good’s coverage	0.5	0.0	0.6	0.1	0.7	0.1
Inverse simpson	181.4	87.4	130.5	77.0	106.0	92.4
*SES.mpd*	0.347	0.337	-0.244	0.850	-0.148	0.730
*SES.mntd*	-0.615	0.611	-0.757	0.554	-0.483	0.755
Time points (n)	6		16		9	

### Community Structure (Beta Diversity) Correlates with Time of Year

Comparison of microbial community structure between samples by principal coordinate analysis revealed five distinct clusters of communities that approximated sampling location and time (**Figure 4B**). Cluster one contained samples associated with the terminus (site 4) and portions from the lower glacier outflow (site 3) that corresponded to the later sampling times (days 194–206). Cluster two consisted of the early outflow samples. Final clusters were from Lake Thomas and separated by sampling location (site 1 vs. site 2) and time of sampling (**Figure 4C**). Time of sampling was a large determining factor for microbial community separation, as observed by plotting PCoA axis 1 against time (**Figure 4C**). No such correlation between time and PCoA axis two was observed (**Figure 4D**).

### Phylogenetic Similarity Amongst Community Members Exhibits Day-to-Day Variability

Community structure, measured by phylogenetic distance with SE size of mean pairwise distance (*SES.mpd*, a.k.a. -1*NRI) and mean nearest taxon distance (*SES.mntd*, a.k.a. -1*NTI), displayed no significant pattern (**Table 1** and Supplementary Figure S4). Most values of *SES.mpd* were not significant (>2 or < -2) and the mean of all values was near zero (-0.1), indicating that nearly all of the microbial communities sampled shared similar phylogenetic distance to null communities independent of sampling location (Supplementary Figure S4A). Similarly, most *SES.mntd* values were negative (mean: -0.65) but were not significantly clumped (<-2; Supplementary Figure S4B). While microbial communities exhibited some degree phylogenetic clumping (e.g., a greater abundance of similar species than expected), it was not significantly different from null constructed communities. While no single sampling site exhibited significant over or under dispersion, phylogenetic distance was observed to vary over several days from samples taken at the terminus.

### Community Structure Correlates with Geochemical Factors Independent of Time

Relationships between patterns of microbial community structure (beta diversity) and geochemistry (pH and conductivity, alkalinity, Na, Mg, Al, Ca, K, Fe, K:Na ratio, and (Mg+Ca):(K+Na)) were assessed by mantel and adonis tests. Mantel tests identified five primary geochemical factors, conductivity (*P* = 0.002, *r*^2^ = 0.135), alkalinity (*P* = 0.038, *r*^2^ = 0.034), sodium (*P* = 0.001, *r*^2^ = 0.408), potassium (*P* = 0.047, *r*^2^ = 0.043), and K:Na ratio (*P* = 0.001, *r*^2^ = 0.23), that were significantly correlated with community structure. These correlations were significant but in most cases correlation coefficients were low. Dispersion of beta diversity, which measures the homogeneity within a given factor around its centroid (here representing sampling location), indicated that variation within each sampling location was not significantly greater than expected from the null hypothesis. Significant differences between the glacial terminus and river centroids were detected (*P*_adjusted_ = 0.02, quantified by Tukey *post hoc* test from betadisper output), confirming that sampling location may impact geochemical correlations. Thus, adonis models with sampling location as a blocking factor were used to further assess correlations between geochemistry and community structure. Sodium was the only geochemical factor significantly correlated with community structure when controlling for sampling location.

## Discussion

Glaciers are unique ecosystems that are highly dynamic and subject to extreme fluctuations in physical and hydrochemical conditions, especially when transitioning into peak melt season. Previous work in these cold environments has demonstrated that microbial communities are diverse and active (Skidmore et al., 2005; Hamilton et al., 2013). However, temporal dynamics of the glacier associated microbiome as it responds to continually changing physical and chemical environment through the melt season are less understood. Thus, we used a culture independent approach to investigate the temporal variability of microbial communities in melt water actively discharged from the terminus of LCG. Because of the inaccessibility of the glacial terminus in the early season due to heavy snow fall and unseasonably cool temperatures, sampling occurred over three distinct but interconnected sampling environments: the proglacial lake Thomas, feeder stream, and terminus discharge. While not anticipated initially, this sampling regime provided insight into spatial differences of microbial communities associated with snowfall, glacial impacted waters, and subglacial environment. Furthermore, because we were routinely sampling from the stream and glacial terminus, we were able to measure the temporal dynamics of microbial community structures.

At high taxonomic levels (phylum and class), the composition of microbial communities was quite similar across all samples, despite the differences in location, geochemistry, and potential redox state of the waters [subglacier waters can be highly anaerobic (Boyd et al., 2010)], and even at finer taxonomic levels the most abundant OTUs recovered from all three sampling sites were present in over 95% of all samples (**Figure 3**). These observed taxonomic linkages between sampling site and time indicates a degree of interconnectivity within the greater glacial ecosystem. Many of the most abundant OTUs display remarkably high similarity to sequences retrieved from other glacial environments (**Figure 3**). While physiological function of OTUs is difficult to infer from 16S rRNA genes, several OTUs are tightly associated with microbial lineages known for performing specific physiological functions. Particularly relevant to glacier biogeochemistry is OTU990, which displays 96% similarity to a known iron-oxidizing microorganism (Weiss et al., 2007) and 100% sequence similarity with an OTU from Bench Glacier (Skidmore et al., 2005), also located in South East Alaska. Enhanced iron liberation from subglacial sediment and water incubations with and without the presence of microbial consortia indicates active iron oxidizing populations (Montross et al., 2013). Thus, microbially mediated iron oxidation is likely ubiquitous and environmentally important. Glaciers are important sources of iron (Raiswell et al., 2006, 2008; Wadham et al., 2010; Bhatia et al., 2013) and transport of oxidized iron to marine environments could enhance biological carbon fixation (Boyd et al., 2007; Statham et al., 2008; Moore et al., 2013). The reoccurring presence of iron oxidizing microorganisms in glacial outflow waters implies biological iron oxidation is an important, ubiquitous glacial process. Further study is needed to fully understand the quantitative effect of iron-oxidizing bacteria, especially at coastally located glaciers such as the LCG.

While the investigation of microbially mediated weathering processes is beyond the scope of the current study, it is evident from the phylogenetic relatives of taxa recovered and the patterns of microbial diversity that subglacial geochemistry and microbial community function are putatively linked. Our data suggests an abundance and ubiquity of recognized lithotrophs that can derive energy in part from the oxidation of iron (Gallionella, Siderooxydans, Thiobacillus, Acidiferrobacter), sulfur (Thiobacillus, Espsilonproteobacteria), and hydrogen (e.g., acetogenesis and methanogenesis by Clostridia and Methanomicrobiales), all of which can be derived from primary weathering of subglacial bedrock. These inorganic energy sources drive chemosynthetic primary production (Boyd et al., 2014), which in turn fuels metabolisms such as heterotrophic respiration (many groups) and fermentation (Bacteroidetes), thus potentially structuring the entire microbial community.

Patterns of community similarity show that communities being ejected from the glacial terminus, while not completely uniform, are more similar to each other than to the lower glacial outflow stream (early samples) and quite different from Lake Thomas (**Figure 4**). Geochemistry of these locations was also different, as Lake Thomas had elevated concentrations of all solutes relative to the glacier terminus. Interestingly, although terminus outflow communities exhibited a unique community signature, the composition of subglacial communities were not significantly phylogenetically dispersed nor clumped when compared to null communities artificially constructed from all datasets (Supplementary Figures S4A,B). This indicates that the proglacial lake, transition stream, and subglacial outflow communities share a large degree of phylogenetic similarity, and that differences in abundance of OTUs contribute significantly to community level differences (bray distances). Thus, shifts in community structure likely originate from environmental changes in pH, salinity, redox potential, or light availability but could also be influenced by secondary microbial inputs from snow melt (Stibal et al., 2012), runoff from proto-soils and atmospheric deposition (Stres et al., 2013) or carbon flux from cyanobacteria and algae associated with snow surface (Stibal et al., 2012). Hamilton et al. (2013) also identified similar phylogenetic connectivity in microbial community structure from cryoconites, subglacier, and surface snow. Suggesting that supra-, en-, and subglacial microbial communities are potentially linked.

It is known that subglacial linkages occur via progression in connectivity within the glacial hydrological network (Tranter et al., 1997). Considering that subglacial water retention time will have a direct influence on the rate and extent of chemical weathering (Anderson et al., 1997), geochemistry of the ejected waters may give insight into the source of waters at LCG. Geochemical characterizations of the ejected waters indicates that Na concentration, which is likely sourced from marine deposition, had decreased significantly over time and that di- to mono- valent cation ratios had increased. Furthermore, Stevenson et al. (under review) measured increases in conductivity, bulk strontium and radiogenic strontium over time. This would suggest that the mixing ratio of surface to sub-glacial waters likely changed over time. Concomitantly, we observed microorganisms sourced from both low residence time waters (e.g., waters moving through main channels connected to the surface, chloroplast DNA **Figure 3** OTU-44217), and high residence time waters (e.g., waters moving through the distributed drainage systems, anaerobic microorganisms such as SR1; see Fountain and Walder, 1998). Finally, at the community level, we saw day-to-day fluctuation in *SES.mntd*, *SES.mpd* and phylogenetic diversity, further indicating that subglacial mixing is not uniform and community composition is dependent upon the mixing ratio of surface water to the englacial and ultimately subglacial environment.

In line with these observations, we saw several punctuated decreased phylogenetic diversity (**Figure 4A**) and punctuated increases of phylogenetic dispersion that corresponded to increases in water discharge volume from the terminus (specifically days 216–218 and 248–249, Supplementary Figure S3A). This was immediately followed by increased phylogenetic clumping in which all but one sample, day 249, is highly phylogenetically clumped (Supplementary Figure S4). These data suggest that water movement, volume and mixing are highly important to influencing the observed microbial communities during these events. At the beginning of the first event, ample subglacial water mixing, which could incorporate several distinct microbial sources, generated communities with greater than expected phylogenetic distances (more positive *SES.-* mpd and mntd values, Supplementary Figure S4). The immediate increase in microbial community phylogenetic similarity (e.g., more negative *SES.- mpd* and *mntd* values) indicate waters from the tail of the discharge contain OTUs that are more similar to one another than expected. This would imply that subglacial water residence times have a large impact on structuring microbial communities by allowing sufficient time to pass for some mechanism of community assembly to occur (Leibold et al., 2004). Higher-resolution sampling (e.g., hourly) would provide deeper insights into such dynamics.

## Conclusion

Glacier ecosystems are extremely important yet transient geological features that link the lithosphere, atmosphere and hydrosphere, including both marine and freshwater systems (Boulton, 2009). This study extends previous work on the glacial microbiome by showing that microbial communities in LCG glacial discharge are remarkably stable through the melt season, where changes in composition reflect shifts in the relative abundance of OTUs that are correlated with geochemistry. Many of the abundant microorganisms of LCG are closely related to organisms from other glacial environments and are putatively involved in geochemically relevant chemolithoautotrophic metabolisms such as iron oxidation. The similarity between spatially isolated glaciers is intriguing, and suggests that microbially driven weathering processes may be pervasive and driving the similarity in key community members.

## Conflict of Interest Statement

The authors declare that the research was conducted in the absence of any commercial or financial relationships that could be construed as a potential conflict of interest.
