# Supplementary material for: Microbial communities of the Lemon Creek Glacier show subtle structural variation yet stable phylogenetic composition over space and time
Source: Front Microbiol. 2015 May 20;6:495. doi: 10.3389/fmicb.2015.00495 (PMC4438255; doi:10.3389/fmicb.2015.00495)

Supplementary Information:

Microbial communities in glacial discharge exhibit spatial and temporal stability and correlate with geochemistry

Cody S. Sheik[1*], Emily I. Stevenson[1], Paul Den Uyl[1], Carli A. Arendt[1], Sarah M. Aciego[1], and Gregory J. Dick[1,2,3]

[1]Department of Earth and Environmental Sciences, [2]Ecology and Evolutionary Biology, [3]Center for Computational Medicine and Bioinformatics, University of Michigan, Ann Arbor, MI, 48109-1004, USA.

*Corresponding author: Cody S. Sheik, csheik@umich.edu

SI Figure 1. Geochemical measurements from LCG over time. Colors represent location of sampling; red = lake, blue = glacial outflow stream, and green = glacial toe. Missing measurements for pH and discharge are represented by zero values.

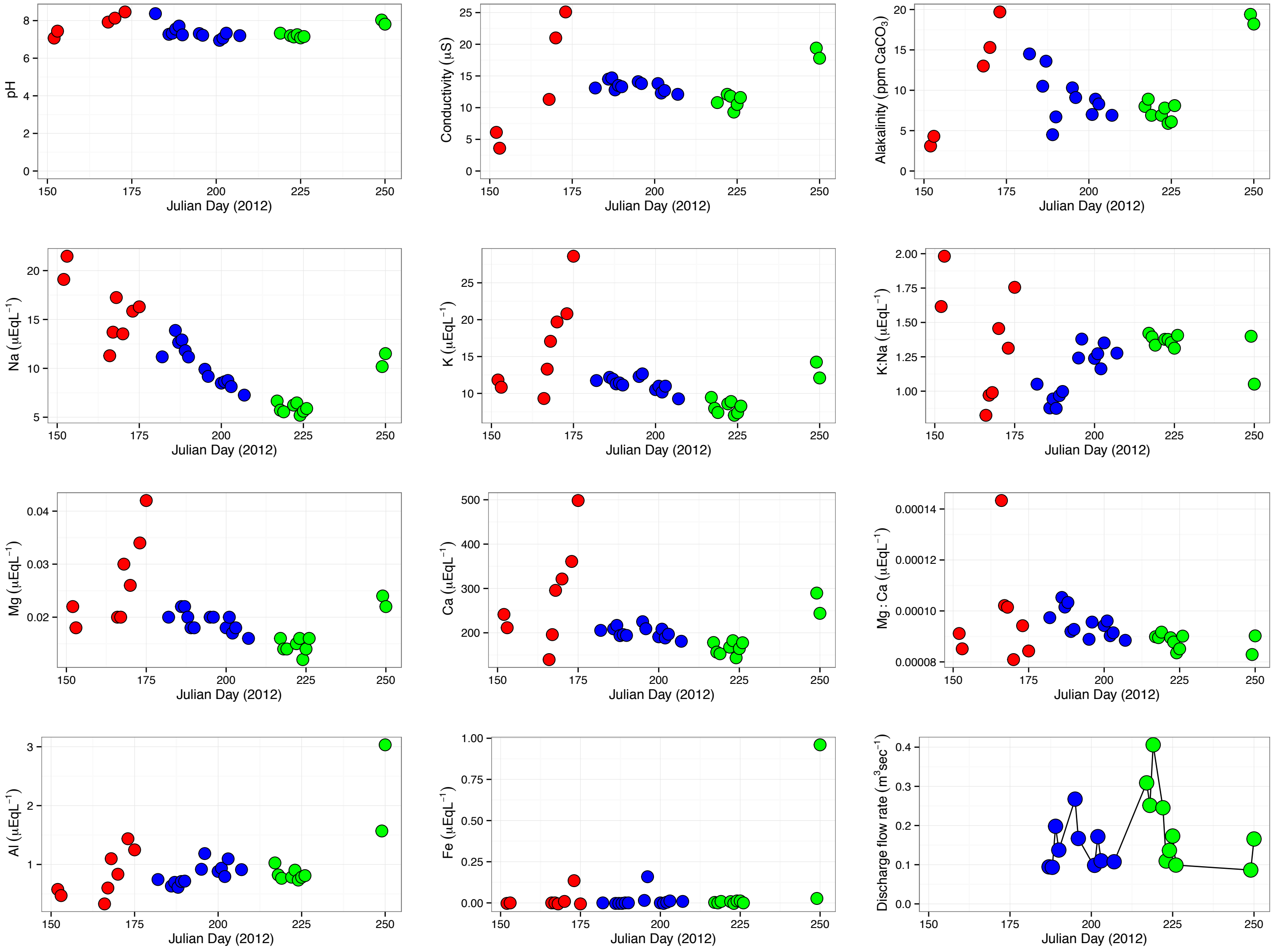

SI Figure 2. Box plot representation of geochemical concentrations as approximated by sampling location. Significance was determined by ANOVA and letter designations of significance were calculated by a Tukey post-hoc significance test.

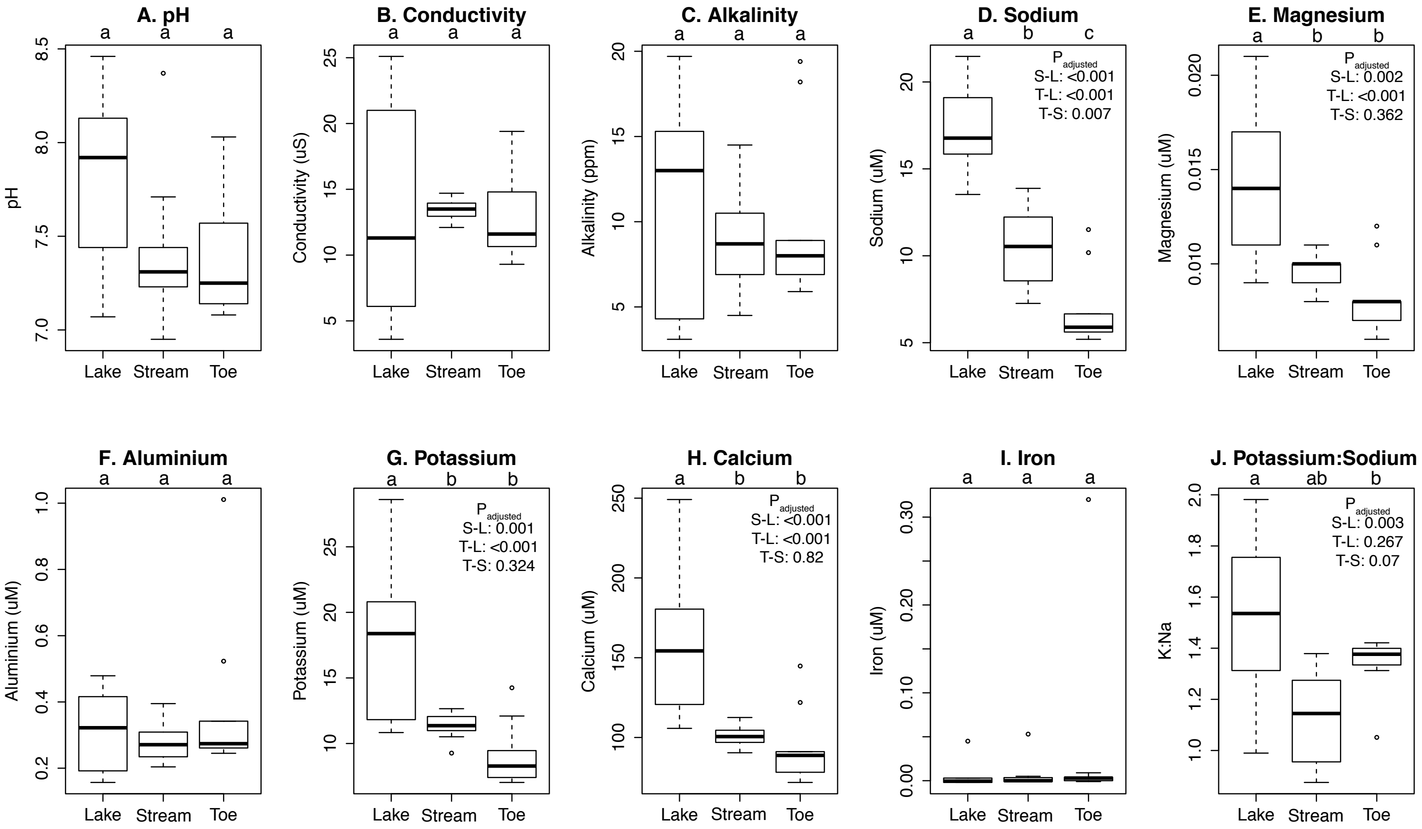

SI Figure 3. Discharge rate (A) and cation ratios of mono valent (B), di-valent (C) and mono to di-valent ratio (D) from LCG outflow waters. Red circles represent the proglacial lake, blue is the outflow stream, and green outflow from the toe.

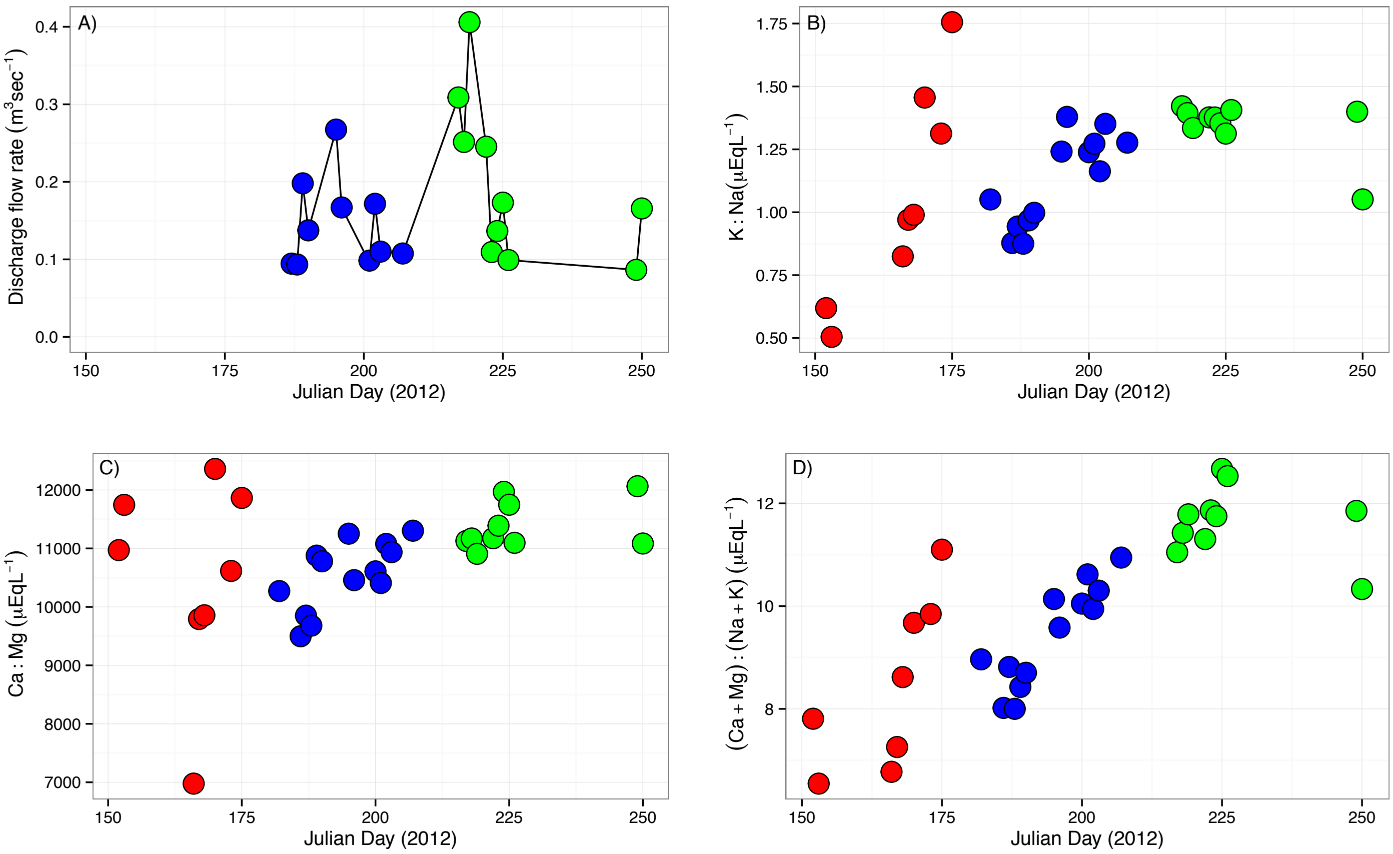

SI Figure 4. Temporal variation of intra-community OTU similarity and assesment of phylogenetic eveness against null communities. Calculation of the mean pairwise distance (A) SES.mpd (-NRI) and mean nearest taxon distance (B) SES.mntd (-NTI). Each sample was subsampled 999 times and compared to null communites to generate signifcance. *SES.mpd* and *SES.mntd*, which are equivalent to -1 times net relatedness index and nearest taxon index (Webb et al., 2008), quantifies the phylogenetic distance between community members from a single sample and compares these values to randomly constructed communities. For both *SES.mpd* and *SES.mntd*, increasingly positive values indicate a phylogenetically dispersed community (*e.g.* contains OTUs that are broadly distributed across a phylogenetic tree), while increasingly negative values indicate a phylogenetically clumped community (*e.g.* community is comprised of very similar OTUs). While both measures calculate similar traits of the community, *SES.mpd* is thought to be more sensitive to phylogenetic clustering over the entire phylogenetic tree while *SES.mntd* is sensitive to localized clumping near the tips of the phylogenetic tree (Kembel, 2009). Significance of *SES.mpd* and *SES.mntd* was determined by comparing the distances of individual communities to distances of randomly selected communities and null communities selected by independent swap method.

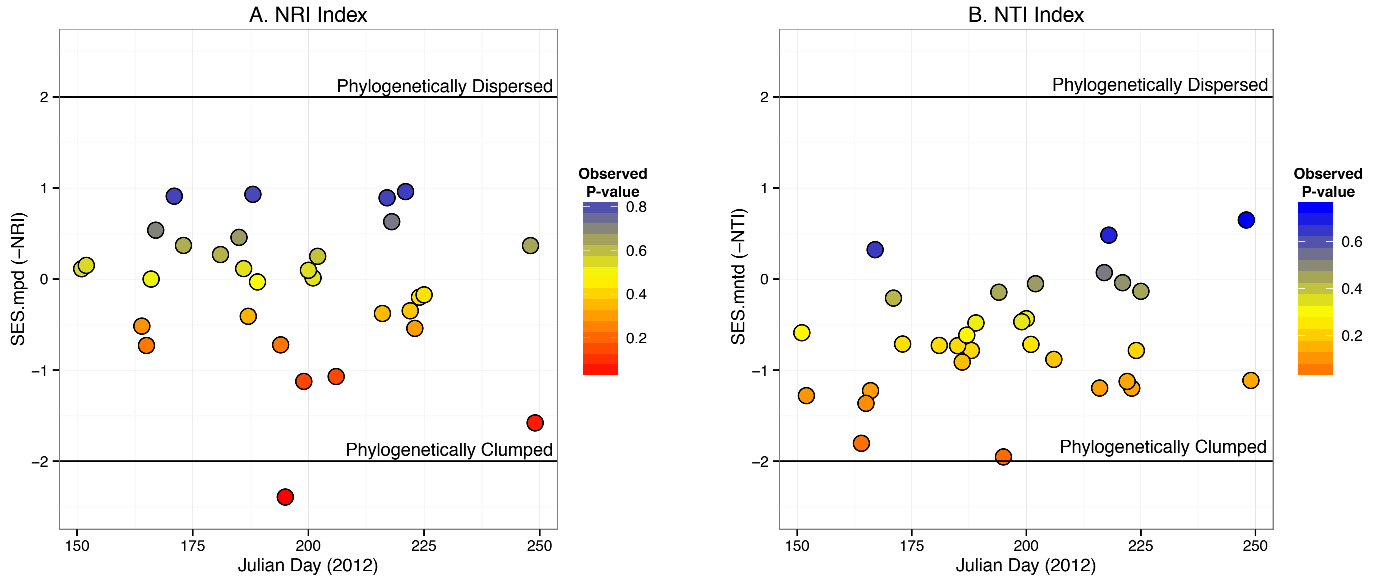

SI Figure 5. Significant geochemical measures correlated with community structure. Geochemical concentrations correlated with community structure were identified by adonis tests. Correlation coefficients and p values are represented in Table 2. PCoA axes one (A) and two (D) reflected over time and color-coded by sampling location and (B, C, E and F) mirrors the community structure from panel A and B but now are color coded by geochemical measurements or concentration.

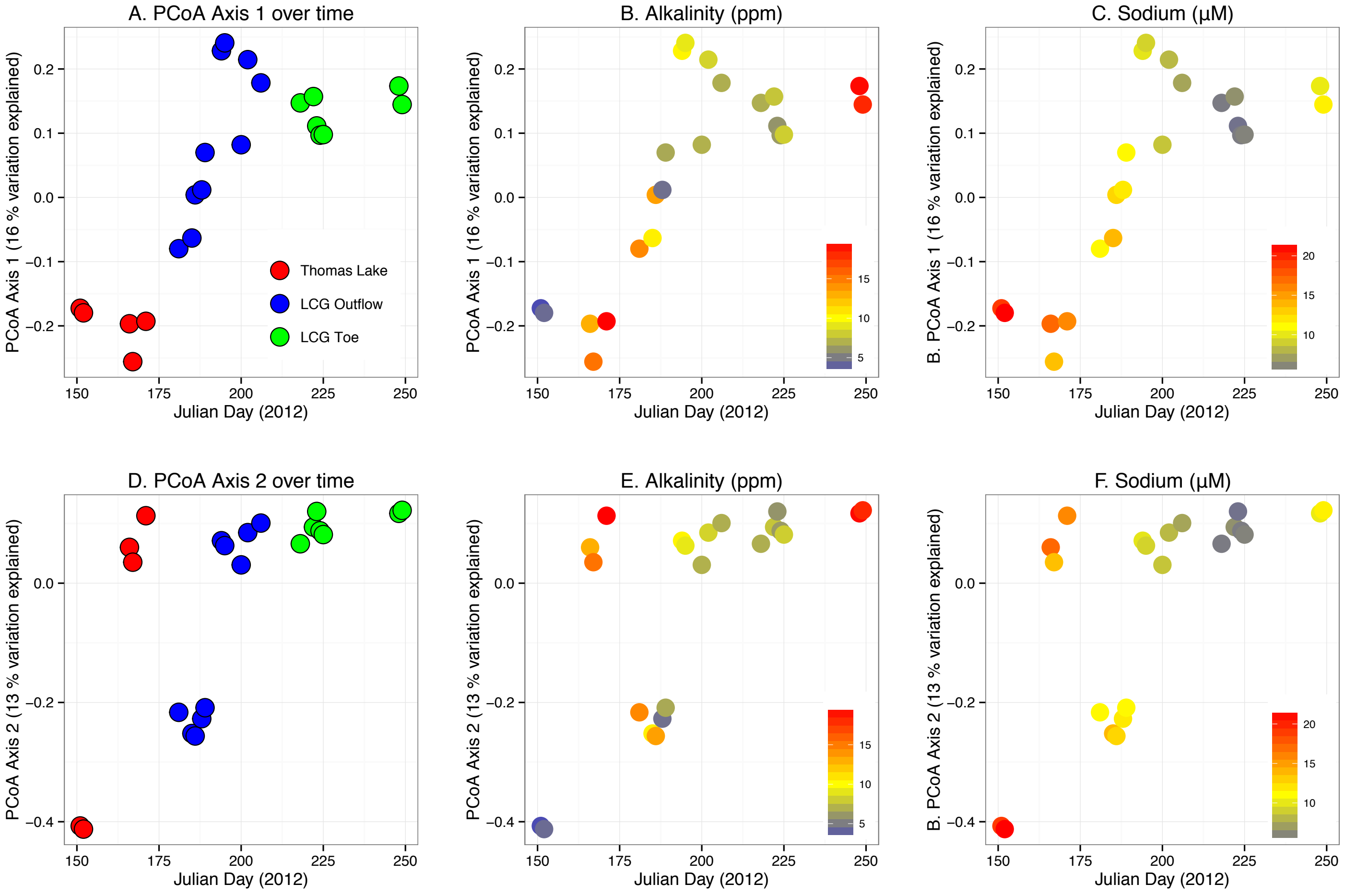

Supplement: Supplementary file 1 [file Data_Sheet_1.PDF]
